# Simplified CRISPR tools for efficient genome editing and streamlined protocols for their delivery into mammalian cells and mouse zygotes

**DOI:** 10.1016/j.ymeth.2017.03.021

**Published:** 2017-03-27

**Authors:** Ashley M. Jacobi, Garrett R. Rettig, Rolf Turk, Michael A. Collingwood, Sarah A. Zeiner, Rolen M. Quadros, Donald W. Harms, Paul J. Bonthuis, Christopher Gregg, Masato Ohtsuka, Channabasavaiah B. Gurumurthy, Mark A. Behlke

**Affiliations:** a Integrated DNA Technologies, Inc., Coralville, IA 52241, USA; b Mouse Genome Engineering Core Facility, Vice Chancellor for Research Office, University of Nebraska Medical Center, Omaha, NE 68198, USA; c Department of Neurobiology & Anatomy, University of Utah School of Medicine, Salt Lake City, UT 84132, USA; d Department of Human Genetics, University of Utah School of Medicine, Salt Lake City, UT 84132, USA; e Department of Molecular Life Science, Division of Basic Medical Science and Molecular Medicine, School of Medicine, Tokai University, Kanagawa 259-1193, Japan; f Center for Matrix Biology and Medicine, Graduate School of Medicine, Tokai University, Kanagawa 259-1193, Japan; g The Institute of Medical Sciences, Tokai University, Kanagawa 259-1193, Japan; h Developmental Neuroscience, Munroe Meyer Institute for Genetics and Rehabilitation, University of Nebraska Medical Center, Omaha, NE 68198, USA

**Keywords:** Genome editing, CRISPR, Cas9, crRNA-tracrRNA, Ribonucleoprotein (RNP) complex, Homology directed repair (HDR)

## Abstract

Genome editing using the CRISPR/Cas9 system requires the presence of guide RNAs bound to the Cas9 endonuclease as a ribonucleoprotein (RNP) complex in cells, which cleaves the host cell genome at sites specified by the guide RNAs. New genetic material may be introduced during repair of the double-stranded break via homology dependent repair (HDR) if suitable DNA templates are delivered with the CRISPR components. Early methods used plasmid or viral vectors to make these components in the host cell, however newer approaches using recombinant Cas9 protein with synthetic guide RNAs introduced directly as an RNP complex into cells shows faster onset of action with fewer off-target effects. This approach also enables use of chemically modified synthetic guide RNAs that have improved nuclease stability and reduces the risk of triggering an innate immune response in the host cell. This article provides detailed methods for genome editing using the RNP approach with synthetic guide RNAs using lipofection or electroporation in mammalian cells or using microinjection in murine zygotes, with or without addition of a single-stranded HDR template DNA.

## 1. Introduction

CRISPR/Cas (clustered regularly interspaced short palindromic repeats/CRISPR-associated) is a bacterial/archaeal immune system that can be adapted to perform sequence-specific genome engineering in mammalian cells and to make novel model organisms [1]. The CRISPR toolbox consists primarily of two components; a guide RNA and a Cas9 nuclease, with a repair DNA template as an optional third component. The guide RNA provides sequence specificity and targets the Cas9 nuclease to a complementary site in the genome where the nuclease creates a double-stranded break. The double-stranded break is healed by cellular repair machinery (non-homologous end joining, or NHEJ), which is often imprecise and can disrupt the amino acid coding sequence if the guide targets a coding exon. The guide RNA occurs naturally as a 2-molecule complex comprising a target-specific crisprRNA (crRNA) bound to a trans-activating crRNA (tracrRNA) that directs binding of the RNAs to Cas9. Alternatively, the guide can comprise a single molecule that is a fusion between the crRNA and the tracrRNA, called a single guide RNA (sgRNA) [2]. Furthermore, novel genetic material can be inserted at the cleavage site by supplying a DNA template, which can be as simple as single-base mutagenesis or insertion of kilobases of new DNA content via homology-directed repair (HDR).

The CRISPR toolbox is being constantly improved. Early methods expressed Cas9 and the guide RNA from plasmid or viral templates, but overexpression of these components from such sources can lead to a high incidence of undesired off-target effects. In this case, double-stranded breaks occur at sites in the genome that are not identical to the guide sequence but that have sufficient homology to enable Cas9-mediated cleavage [3]. Direct delivery of the Cas9 nuclease with guide RNA as a ribonucleoprotein (RNP) complex limits the amount of the critical components and gives a “fast on/fast off” character to the genome editing machinery, resulting in a significant reduction in off-target effects [4–6]. Two different versions of Cas9 RNP complexes can be employed: (1) combination of sgRNA and Cas9 protein, and (2) combination of crRNA, tracrRNA (two separate strands to form a complete guide RNA) and Cas9 protein. Cas9, the common component of the two versions, is used as a recombinant protein; the RNA components in the first version (sgRNA) are typically synthesized using *in vitro* transcription and the RNA components in the second version (crRNA and tracrRNA) are chemically synthesized. To distinguish the two types of RNPs, we recently proposed the terms sgRNP and ctRNP for the complexes containing sgRNA or crRNA + tracrRNA as RNA components respectively [7].

Until recently, only two types of DNA repair templates have been used: (1) a single-stranded synthetic oligodeoxynucleotide (ssODN) if the aim is to insert or modify a short sequence (up to 200 bases, usually with 30–60 base homology arms) [8–10], or (2) a double-stranded DNA (dsDNA) with much longer homology arms (500–1000 bases) that supports insertion of up to several thousand bases [11]. However, recent reports have demonstrated that long single-stranded DNAs (ssDNAs) enzymatically generated from cloned sources can be used as repair templates that do not require as long of homology arms yet can show higher efficiency of insertion than similar templates in dsDNA form [12,13]. The same RNP protocols can be used for both sgRNP and ctRNP complexes, with the exception that the crRNA and tracrRNA must be annealed before final complex formation for the ctRNPs.

In this report, we describe methods and protocols related to use of CRISPR RNPs containing chemically-modified crRNA + tracrRNA complexed with Cas9 protein for direct delivery into cells and mouse zygotes. Specifically, we provide protocols for (1) lipofection of ctRNPs into mammalian cells, (2) electroporation of ctRNPs into mammalian cells, (3) general outline of genotyping and screening for mutations, and (4) microinjection of ctRNPs and long ssDNA donors into mouse zygotes for creating knock-in alleles. These streamlined protocols are suitable for delivering either ctRNPs or sgRNPs with optional repair DNAs.

## 2. Methods

### 2.1. Ribonucleoprotein complex lipofection

**Fig. 1:**
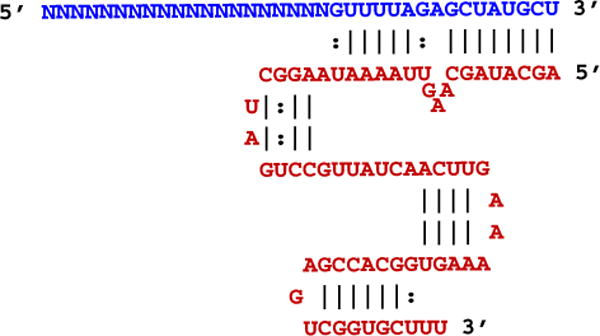
Aligned crRNA and tracrRNA sequences in the guide RNA complex. The crRNA is shown (blue) aligned with the tracrRNA (red). The variable target-specific protospacer domain of the crRNA is indicated with “N” bases.

**Fig. 2:**
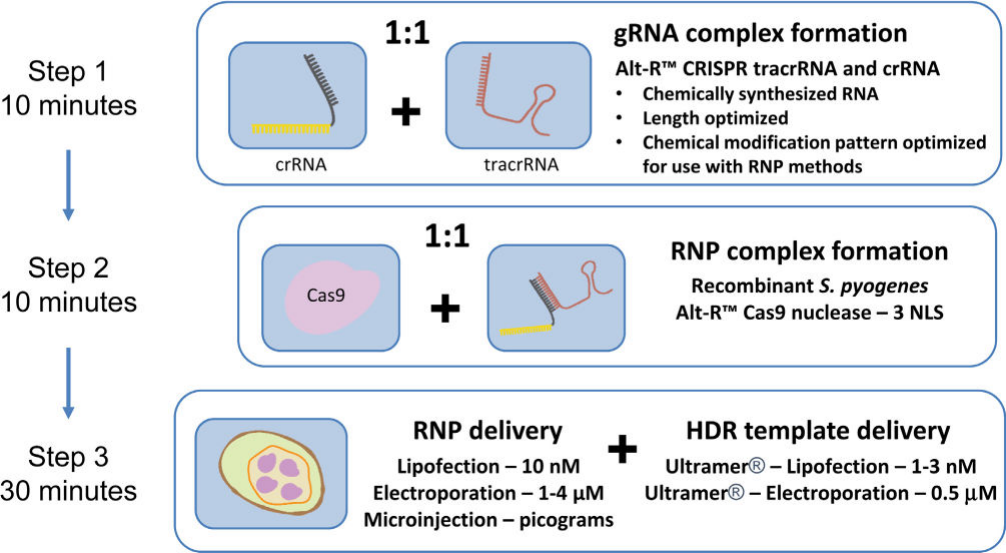
Genome editing workflow using the ctRNP approach. The steps of crRNA:tracrRNA annealing, RNP complex formation with recombinant Cas9 protein, and cell delivery are schematically outlined. (reprinted with permission from Integrated DNA Technologies, Inc.)

All methods described herein employ a CRISPR system that uses two synthetic RNA oligonucleotides, a crRNA and a tracrRNA, that must be annealed prior to mixing with Cas9 protein and subsequent delivery as a ctRNP complex. Further, the RNAs employed are chemically-modified and length optimized variants of the native guide RNAs (Alt-R™ CRISPR crRNAs and tracrRNA, Integrated DNA Technologies, Coralville, IA, USA). The optimized lengths of crRNA and tracrRNA are 36 and 67 bases respectively (Fig. 1). Lipofection is the least expensive method for introducing Cas9 RNP into cell lines amenable to lipofection. The present protocol has been optimized for delivery into HEK293 cells. Electroporation (Section 2.2) may be considered to introduce RNP into cell lines or cell types where lipofection is not efficient. Cas9 ctRNP lipofection can be coupled with co-transfection of ssODNs as HDR templates. When a ssODN HDR donor is included, we suggest use of high-fidelity Ultramer^®^ DNA oligonucleotides (Integrated DNA Technologies) for templates of up to 200 bases and suggest using desalted oligonucleotides (PAGE purification adds cost and, in some settings, toxicity from residual acrylamide or urea with this method of preparation). We recommend adding 30–50 base homology arms on either side of the predicted crRNA cleavage site. The basic protocol involves 3 steps: 1) annealing the crRNA and tracrRNA to form a complete guide RNA, 2) forming a complex between Cas9 and the guide RNAs, and 3) delivery into cells (Fig. 2).

#### 2.1.1. Lipofection of ctRNP complexes for NHEJ into HEK293 cells

1. Form guide RNA complexes by combining the crRNA and tracrRNA in equal molar amounts in IDT Duplex Buffer (30 mM HEPES, pH 7.5, 100 mM Potassium Acetate) at 1 μM concentration by heating the oligos to 95 °C and slowly cooling to room temperature. We typically keep working stocks of crRNAs and tracrRNA at 10 μM concentration in TE (10 mM Tris, pH 7.5, 0.1 mM EDTA), in which case mix 1 μL of crRNA and 1 μL of tracrRNA with 8 μL of Duplex Buffer. While not always necessary, the heat/cool step improves performance for approximately 10% of target sites. Excess of the 1 μM crRNA:tracrRNA complex can be stored for later use at 4 °C, −20 °C or −80 °C for at least 3 months. Aligned sequences of the crRNA:tracrRNA complex after annealing are shown in Fig. 1.
2. Dilute Alt-R™ 3NLS Cas9 Nuclease (Integrated DNA Technologies) from stock 61 μM (10 mg/mL) to 1 μM in Opti-MEM (Thermo Fisher Scientific, Carlsbad, CA USA). Final transfections will employ 10 nM ctRNP complex.
3. The following preparation of the ctRNP complex is intended for biological triplicates in 96-well culture format (3.5× of the required solution for 1 well is made using this protocol):
  - The ctRNP complex is prepared by combining 5.25 μL of the 1 μM crRNA:tracrRNA complex with 5.25 μL of the 1 μM diluted stock of Cas9 protein. (Note: excess of the 1 μM RNP complex can be made and stored for later use at 4 °C or −80 °C for at least 3 months.)
  - Add 77 μL of Opti-MEM medium, bringing the final volume to 87.5 μL, yielding a final 60 nM concentration of RNP complex.
4. Incubate this mixture at room temperature for 5 min.
5. Mix 4.2 μL of Lipofectamine^®^ RNAiMAX (Thermo Fisher Scientific) with 83.3 μL of Opti-MEM and add this mixture to each sample of ctRNP complex (87.5 μL), resulting in a final volume of 175 μL with an RNP concentration of 30 nM.
6. Incubate RNP-lipid complexes at room temperature for 20 min. The ctRNP transfection solution is now ready for use.
7. Trypsinize and count HEK293 cells cultured in Dulbecco’s Modified Essential Medium (DMEM) supplemented with 10% fetal bovine serum (FBS) (Thermo Fisher Scientific). Pellet HEK293 cells. Due to the frequent presence of RNases in trypsin, which can degrade the guide RNAs, cells should be washed after trypsinization with 1X PBS (phosphate buffered saline). This wash step is critical with electroporation and is also recommended for lipofection. Resuspend cells in DMEM + 10% FBS at 40,000 cells per 100 μL.
8. Aliquot 50 μL of ctRNP transfection solution followed by 100 μL cell suspension to 3 wells in a 96-well tissue culture plate to create biological triplicates (reverse transfection format). The final concentration of the ctRNP complex in the transfection is 10 nM with 1.2 μL RNAiMAX used per well.
9. Incubate cells for 48–72 h at 37 °C and 5% CO_2_ before isolating genomic DNA to assess genome editing results (Section 2.3 below).

#### 2.1.2. Lipofection of ctRNP complexes with ssODNs for HDR

1. Prepare DNA HDR template by diluting stock ssODN Ultramer^®^ (Integrated DNA Technologies) to 0.3 μM in TE.
2. Form Cas9 ctRNP complexes as described above in *Lipofection of ctRNP Complexes for NHEJ* through Step 3b, using 71.75 μL of Opti-MEM (instead of 77 μL). Incubate 5 min at room temperature. Then add 5.25 μL of the HDR ssODN to give a final concentration of 60 nM RNP complex and 18 nM of HDR oligonucleotide in 87.5 μL.
3. Proceed with steps 6–9 as outlined above (Section 2.1.1); this results in a reverse transfection of 10 nM ctRNP complex and 3 nM HDR oligonucleotide per well with 1.2 μL RNAiMAX and 40,000 HEK293 cells in 150 μL total transfection volume.

### 2.2. Electroporation of Cas9 ctRNP complexes

RNP delivery via electroporation is the preferred methodology for hard-to-transfect cell lines and primary cell cultures. Here, we describe the transfection of HEK293 cells using the Amaxa^®^ Nucleofector^®^ system (Lonza, Basel, Switzerland), and the transfection of Jurkat cells using the Neon™ Transfection System (Thermo Fisher Scientific). For both methodologies, we recommend the use of cells with low passage numbers. Furthermore, cells should be subcultured 2–3 days before electroporation and diluted appropriately to result in an optimal confluency of 80–90% on the day of electroporation. Delivery of RNP by electroporation requires relatively high concentrations of RNP compared to lipofection. Addition of the Alt-R™ Cas9 Electroporation Enhancer reagent (which is a single-stranded oligonucleotide with no homology to human, mouse, or rat genomes) during electroporation increases efficiency of *indel* formation through stimulation of error-prone repair pathways (“Non-homologous Oligonucleotide Enhancement”, or “NOE” [14]) and possibly also by improving RNP uptake; the magnitude of benefit varies with the electroporation protocol and cell type. We have not found any disadvantage from use of the Electroporation Enhancer and thus include it in all electroporation experiments.

#### 2.2.1. Electroporation using the Amaxa^®^ Nucleofector^®^ (Lonza), into HEK293 cells

1. Subculture HEK293 cells 2–3 days before electroporation resulting in a confluency of 80–90% on the day of electroporation.
2. Form guide RNA complexes by combining the crRNA and tracrRNA in equal molar amounts in IDT Duplex Buffer (30 mM HEPES, pH 7.5, 100 mM potassium acetate) at 100 μM concentration by heating the oligos to 95 °C and slowly cooling to room temperature.
3. Prepare a 96-well cell culture plate to receive cells following electroporation. Pre-warm antibiotic-free culture media (DMEM + 10% FBS) by filling the desired number of wells (each experimental condition should be tested in triplicate) with 175 μL of the media. Also, pre-warm additional media (75 μL per experimental condition) in a separate sterile tube, for resuspending electroporated cells. Keep both in a tissue culture incubator (37 °C, 5% CO_2_).
4. Harvest cells by trypsinization, and neutralize by adding culture media which contains FBS. Triturate to achieve single-cells in suspension.
5. Count cells. For a single cuvette in a 96-well Nucleofection Module, we typically use 3.5 × 10^5^ HEK293 cells. Transfer total number of cells needed to a sterile, 15 mL tube.
6. Centrifuge the cells at 10*g* for 10 min at room temperature. Aspirate the supernatant.
7. Resuspend the cells in 5 mL of 1X PBS. Repeat step 6.
8. Resuspend the cells in the appropriate volume of Nucleofection Solution SF (20 μL of Nucleofection Solution SF per 3.5 × 10^5^ cells).
9. While the cells are being centrifuged (2 × 10 min), prepare the ctRNP.
10. The ctRNP complex is prepared by mixing 1.7 μL of the stock Alt-R™ S.p. Cas9 3NLS protein (which is supplied at 61 μM, so 104 pmol of Cas9 is used) with 130 pmol guide RNA complex (1.3 μL of the 100 μM solution prepared in Section 2.2.1 step 2) with 2 μL of 1X PBS to yield a final volume of 5 μL. This volume is sufficient for a single cuvette in a 96-well Nucleofection Module. Incubate the RNP mixture at room temperature for 10–20 min.
11. Combine 20 μL of cell suspension (step 8) with 5 μL of ctRNP complex (step 10).
12. Prepare a stock solution of the Alt-R™ Cas9 Electroporation Enhancer by adding TE to the lyophilized oligonucleotide at a final concentration of 100 μM (20 μL TE for 2 nmole, 100 μL TE for 10 nmole). Add 1 μL of Alt-R™ Cas9 Electroporation Enhancer (100 μM stock tube) to the RNP complex and mix.
13. The total volume is 26 μL. Therefore, the final concentrations during electroporation are 4 μM Alt-R™ S.p. Cas9 3NLS, 5 μM guide RNA complex, and 4 μM Alt-R™ Cas9 Electroporation Enhancer.
14. Transfer 25 μL of the mixture to a cuvette within the 96-well Nucleofection Module. Place the Nucleofection Module into the Amaxa 96-well Shuttle Device, and electroporate the cells using protocol 96-DS-150. Note that electroporation protocols are cell-type dependent and may need to be varied or optimized for every different cell line used.
15. Add 75 μL pre-warmed media to the cell mixture in the cuvette and gently pipet up and down 2 times. Transfer 25 μL of the electroporated cells to 175 μL pre-warmed media in triplicate (the electroporation product is equally aliquoted into 3 wells providing 3 biological triplicate cultures for downstream analysis).
16. Incubate cells in a tissue culture incubator (37 °C, 5% CO_2_) for 48–72 h before isolating genomic DNA to assess genome editing results (Section 2.3 below).

#### 2.2.2. Electroporation using the Neon™ Transfection System (Thermo Fisher Scientific), into Jurkat cells

1. Subculture Jurkat cells (ATCC TIB-152) 2–3 days before electroporation to result in a final density between 1 × 10^5^ and 1 × 10^6^ cells/mL on the day of electroporation.
2. Form guide RNA complexes by combining the crRNA and tracrRNA in equal molar amounts in IDT Duplex Buffer (30 mM HEPES, pH 7.5, 100 mM potassium acetate) at 100 μM concentration by heating the oligos to 95 °C and slowly cooling to room temperature.
3. Prepare a 96-well cell culture plate to receive cells following electroporation. Fill a well for each experimental condition with 190 μL antibiotic-free culture media for diluting electroporated cells (RPMI + 10% FBS). Additionally, aliquot 150 μL culture media for each experimental condition in triplicate, for final cultures to grow cells after electroporation. Pre-warm in a tissue culture incubator (37 °C, 5% CO_2_).
4. Triturate to achieve a suspension of single-cells. Jurkat cells grow in suspension, so a trypsinization step is not necessary.
5. Count cells. For a single 10 μL Neon™ Tip, we typically use 5 × 10^5^ Jurkat cells. Transfer total number of cells needed to a sterile, 15 mL tube.
6. Centrifuge the cells at 60*g* for 10 min at room temperature. Aspirate the supernatant.
7. Resuspend the cells in 5 mL of 1X PBS. Repeat step 6.
8. Resuspend the cells in the appropriate volume of Buffer R (use 9 μL of Buffer R per 5 × 10^5^ cells).
9. While the cells are being centrifuged (2 × 10 min), prepare the RNP.
10. Prepare a 36 μM working dilution of Cas9 by mixing 3 μL of Alt-R™ S.p. Cas9 3NLS stock (61 μM) with 2 μL Buffer R. Prepare a 43 μM working dilution of guide RNA complex by mixing 4.3 μL guide RNA complex (100 μM) with 5.7 μL Buffer R. The ctRNP complex is prepared by mixing 0.5 μL of the Cas9 working dilution (36 μM) with 0.5 μL of the guide RNA working dilution (43 μM). This 1 μL volume is sufficient for a single 10 μL Neon Tip. Incubate the ctRNP at room temperature for 10–20 min.
11. Combine 9 μL of cell suspension (step 8) with 1 μL of ctRNP complex (step 10).
12. Prepare a stock solution of the Alt-R™ Cas9 Electroporation Enhancer by adding TE to the lyophilized oligonucleotide at a final concentration of 100 μM (20 μL TE for 2 nmole, 100 μL TE for 10 nmole). Prepare a working dilution of the Alt-R™ Cas9 Electroporation Enhancer at 10.8 μM (dilute 10.8 μL of a 100 μM stock solution to a final volume of 100 μL using TE). Add 2 μL of Alt-R™ Cas9 Electroporation Enhancer working dilution (10.8 μM) to the ctRNP complex and mix. Leftover Enhancer can be stored at −20 °C for future use.
13. The total volume is 12 μL. Therefore, the final concentrations during electroporation are 1.5 μM Alt-R™ S.p. Cas9 3NLS, 1.8 μM guide RNA complex, and 1.8 μM Alt-R™ Cas9 Electroporation Enhancer.
14. Aspirate 10 μL of ctRNP/cell suspension using the 10 μL Neon™ Tip, avoiding air bubbles.
15. Electroporate the cells using the Neon™ Transfection System according to the manufacturer’s recommendations. For Jurkat cells, we use the following parameters: pulse voltage – 1600 V, pulse width – 10 ms, and pulse No. – 3.
16. Pipet the electroporated cells into 190 μL pre-warmed resuspension media, and mix gently by pipetting up and down.
17. Transfer 50 μL of resuspended cells in triplicate to culture plate wells containing 150 μL media, which were originally aliquoted in step 3 and should be warm in the incubator (the electroporation product is equally aliquoted into 3 wells providing 3 biological replicate cultures for downstream analysis).
18. Incubate cells in a tissue culture incubator (37 °C, 5% CO_2_) for 48–72 h before isolating genomic DNA to assess genome editing results (Section 2.3 below).

### 2.3. Assays for measuring total editing versus HDR-mediated template insertion – Enzymatic methods and Next-Generation sequencing

Each genome editing experiment will generate a heterogeneous mixture of cells in the transfected population having different editing events at the targeted locus. Some will result from NHEJ where insertion/deletion (*indel*) events dominate. Some will result from HDR and yield “perfect” (desired) results while others will have imperfect HDR alterations. Next generation sequencing (NGS) methods can easily interrogate thousands to millions of events simultaneously and provides the best view of the range of editing that occurred in the cell population. Unfortunately, NGS can be expensive and may have long turnaround times. Simpler, albeit less accurate, enzymatic methods (described below) can serve as inexpensive alternative methods.

Endonuclease mismatch cleavage (EMC) assays to estimate the frequency of *indel* formation are fast and easy to perform. A PCR amplicon is made using primers that flank the crRNA cleavage site and a final denature/anneal step is used to allow formation of heteroduplexes between wild type and mutant DNA strands. The amplicons are incubated with a mismatch endonuclease, such as Surveyor^®^ (Cel I) or T7 Endonuclease I (T7EI), which cleave the duplex at the site of a heteroduplex mismatch bubble. Cleaved fragments are then detected using gel electrophoresis, or other more automated sizing technology [15,16]. EMC assays can underestimate editing efficiency as some single-base events are not cleaved and therefore not detected; also, if a single editing product dominates, those mutant strands can anneal to form homoduplexes that likewise are undetected and therefore read as being wild type. Editing events that lead to very large *indels* may fail to amplify and thus will also not be detected by this approach.

Along the same lines as general EMC assays, introduction of a novel restriction site during HDR creates a restriction fragment length polymorphism (RFLP), and allows for interrogation of the HDR event using enzymatic cleavage by that restriction endonuclease. We demonstrate this by introducing an EcoRI site by HDR, as discussed below. Note, however, that in some sequence contexts *indel* formation during NHEJ can also create a new restriction site in the absence of HDR template insertion and lead to false positive results. Sequencing of the PCR amplified target region can help distinguish true- or false-positive RFLP results. Methods are outlined below to use enzymatic cleavage assays as well as NGS analysis to quantify the rate of total editing efficiency vs. HDR in a cell population. Additional methods are available to assess the mutation outcome following CRISPR/Cas9 cleavage and repair that are not shown in the protocols employed here. For example, Sanger sequencing results can be analyzed using sequence trace decomposition (“TIDE” analysis) [17], fluorescent-labeled primer-extension on an amplicon spanning the Cas9 cut site can be used to map indels using Indel Detection by Amplicon Analysis (IDAA) [18], or high resolution melt analysis (HRM) can be applied to PCR amplicons that span the Cas9 cut site [19].

#### 2.3.1. T7 Endonuclease I cleavage assay

1. CRISPR/Cas9 is used to generate a cell population having NHEJ or HDR gene editing events using the methods in Sections 2.1 and 2.2 above. After 48–72 h incubation, lyse cells by removing media, washing with 100 μL of 1X PBS per well, and adding 50 μL of QuickExtract™ – DNA Extraction Solution (Epicentre, Madison, WI, USA). Process the lysate according to the manufacturer’s recommendations for DNA isolation and dilute the final product 3-fold in nuclease-free water to a final volume of 150 μL.
2. Amplify genomic DNA using PCR primers that flank the crRNA cleavage site and have previously been validated to result in a single, locus-specific amplicon. Typically, we employ 1.5 μL of the cellular DNA (1% of what was produced from a single well of a 96-well plate), which is usually around 15 ng of DNA, in a 10 μL amplification reaction using KAPA HiFi Hot Start DNA Polymerase (Kapa Biosystems, Wilmington, MA, USA). Ideally the primers are positioned 200 bases (or more) on either side of the cleavage site under interrogation. The examples shown below employ a 1083 base amplicon in the human *HPRT1* gene that is made with the primers shown in Table 1 using the PCR program: 95 °C for 5 min + [98 °C for 20 s + 67 °C for 15 s + 72 °C for 30 s] × 30 cycles + 72 °C for 2 min. Anneal temperature should be optimized for each primer set to ensure that a single amplicon is produced for downstream evaluation.
3. Following PCR, add 1.3 μL of 10X NEBuffer 2 (New England Bio-Labs, Ipswich, MA, USA) and 1.7 μL of water, bringing total volume to 13 μL. Form heteroduplexes by denaturing and slowly reannealing the PCR amplicons. We prefer to do this in a PCR thermal cycler, using the program:
  - Ramp Rate 1 – 95–85 °C = −2 °C/s
  - Ramp Rate 2 – 85–25 °C = −0.3 °C/s
4. T7EI is diluted by taking 1 μL (10 U/μL stock) and adding 1 μL 10X NEBuffer 2 and 8 μL water. Digest heteroduplexes with 2 μL of diluted T7EI (2 U) with incubation at 37 °C for 60 min.
5. Cleavage products can be visualized by agarose gel electrophoresis (or any other method that can separate DNA fragments in this size range). For agarose gel visualization, load ~1/2 of the reaction onto the gel.
6. From digitized agarose gel image data, use the relative pixel density in the following formula where F1 refers to fragment 1, F2 refers to fragment 2 and FL refers to the full-length undigested amplicon (Eq. (1)) [20]:
  (1) $$\mathrm{Editing}\;\mathrm{Efficiency}(\text{\%})=100\times \left(1-\sqrt{1-\frac{\left(F1+F2\right)}{\left(F1+F2+FL\right)}}\right)$$
7. We prefer to employ capillary electrophoresis using the Fragment Analyzer™ (Advanced Analytical Technologies, Ankeny, IA, USA). This instrument allows automated processing in 96-well plate format and can provide digital, quantitative analysis of the EMC assay in a 90 min run. Employ the Mutation Discovery Kit (Advanced Analytical Technologies); dilute the 15 μL T7EI cleavage reaction in 100 μL of 0.1X TE and run auto-injection into the Fragment Analyzer™.
8. Using data from the Fragment Analyzer™, which automatically adjusts for relative molar abundance in the output data, editing frequencies are calculated using the following formula (Eq. (2)): average molar concentration of the cleaved products/(average molar concentration of the cleaved products + molar concentration of the full-length product) × 100.
  (2) $$\mathrm{Editing}\;\mathrm{Efficiency}(\text{\%})=100\times \left(\frac{\mathrm{Average}(F1+F2)}{(\mathrm{Average}(F1+F2))+FL}\right)$$

The calculated numbers from EMC assays give an estimate of total gene editing events in the cell population, understanding that this number may be an underestimate as discussed above.

#### 2.3.2. RFLP assay for HDR template insertion

1. Restriction endonuclease cleavage assay can be performed to estimate the efficiency of HDR in a genome editing experiment if the HDR template introduces a new restriction site into the assay amplicon. In the present example, we employ an artificial EcoRI site. Perform CRISPR/Cas9 genome editing with an HDR template as described in Section 2.1.2 above. Isolate genomic DNA and produce PCR amplicons that include the modified locus as described in Section 2.3.1 *steps 1*–*2*, but do not proceed to make heteroduplexes as would be done when performing an EMC assay.
2. Dilute EcoRI (New England BioLabs) from stock taking 1 μL (10 U/μL) and adding 1 μL of 10X CutSmart^®^ Buffer (New England BioLabs), and 8 μL of water. Add 2 μL of the diluted EcoRI mix to the 10 μL PCR reaction (from Section 2.3.1 *step 2*) and incubate for 60 min at 37 °C.
3. Visualize amplicon digestion products on an agarose gel or on the Fragment Analyzer™. EcoRI cleavage efficiency is assessed as outlined in Section 2.3.1 *steps 5*–*7* above.

#### 2.3.3. Next Generation Sequencing – Nextera™ (Illumina^®^) library preparation

Any NGS platform can be used for DNA sequence analysis of genome editing events. Methods provided below employ the Illumina^®^ platform. PCR amplicons can be designed to include the necessary universal sequences for direct input into the NGS process; this approach is best suited to study gene editing events at a single crRNA site per amplicon (1 amplicon, 1 crRNA). The method below employs the Illumina^®^ Nextera™ library construction method where fragmentation of larger amplicons followed by library preparation allows for many sites within a long amplicon to be simultaneously interrogated. This method is suitable for assessing editing of many crRNAs in relatively close genomic proximity (+/− 1000 bp) as well as in identification of large insertions or deletions (>100 bp). Nextera™ library preparation can be used, for example, to evaluate genomic DNA samples from nucleofection and electroporation experiments to evaluate if any incorporation of the ssDNA Alt-R™ Cas9 Electroporation Enhancer occurred. Large insertions are not identified as effectively using traditional amplification-based library preparation methods (see below) due to amplicon sizing steps and sequence read length limitations. NGS starting with long amplicons and Nextera™ library preparation can also be used to simultaneously interrogate pooled amplicons from “PAM site walk experiments”, where crRNAs are tiled through a region of interest to find the most active sites for later use.

1. Generate PCR amplicons flanking the targeted cleavage site using KAPA HiFi Polymerase as described in Section 2.3.1 *step 2* above. In the example shown below, the PCR makes a 673 base human *HPRT1* amplicon using the primers shown in Table 1 with the PCR program: 95 °C for 5 min + [98 °C for 20 s + 67 °C for 15 s + 72 °C for 20 s] × 30 cycles + 72 °C for 2 min. Annealing temperature should be optimized for each primer set to ensure that a single amplicon is produced for downstream evaluation.
2. Purify amplicons by solid phase reversible immobilization (SPRI) bead cleanup using the 0.8X Agencourt Ampure XP (Beckman Coulter, Indianapolis, IN, USA) per the manufacturer’s protocol.
3. Prepare NGS sequencing libraries using the Nextera™ XT Sample Preparation Kit (Illumina^®^) per the manufacturer’s protocol.
4. Perform DNA sequencing on an Illumina MiSeq platform using MiSeq Reagent Kit v2 300 Cycles. Note that use of the Nano Flow Cell can reduce NGS material costs if the number of samples being sequenced in multiplex format is low.
5. Perform data analysis; we employ a freeware computational pipeline for analyzing CRISPR genome editing experiments, CRISPResso [21]. The plain text output from the computational analysis is used to generate graphical reports for visualizing *indel* profiles relative to the wild-type sequences.
6. Additional data analysis can be performed by first doing a soft trim of all reads prior to comparison to a reference sequence using BLAST. From the detailed alignment data, variations relative to the reference sequence are identified and their relative frequencies are calculated.

#### 2.3.4. Next Generation Sequencing – target amplification

Amplification-based sequencing can be used to interrogate editing at one crRNA location with a single amplicon. In this design, the locus-specific primers flank the Cas9 cut site by approximately 75-bp. Primers contain universal “tails” at the 5′-ends that allow for a second amplification step to incorporate Illumina adapter sequences as well as sample-specific barcodes. Gene editing is determined by bioinformatics analysis through alignment of paired-end reads and programmatic evaluation of variants relative to a reference sequence. Identifying the insertions/deletions/substitutions resulting from double-stranded break repair via this method is the easiest and most straightforward approach to use NGS for evaluating gene editing.

1. Generate PCR amplicons flanking the targeted cleavage site using a wild-type *Taq* polymerase. Locus-specific primers for evaluating editing by HPRT1 38087 and 38285 crRNAs are shown in Table 1. Amplicons with 5′-universal “tail” sequences are generated with the following program: 95 °C for 5 min + [95 °C for 15 s + 60 °C for 60 s] × 8 cycles + 99 °C for 15 min. Again, annealing temperature should be optimized for each primer set to ensure that a single amplicon is produced for downstream evaluation.
2. Purify amplicons by SPRI bead cleanup using the 1.5X Agencourt Ampure XP (Beckman Coulter, Indianapolis, IN, USA) per the manufacturer’s protocol.
3. NGS platform-specific adapters are incorporated in a subsequent amplification with universal primers shown in Table 1. Amplicons are again produced with *Taq* polymerase and with the following cycling conditions: 95 °C for 5 min + [95 °C for 15 s + 60 °C for 30 s + 72°C for 30 s] × 18 cycles + 99 °C for 15 min.
4. Purify amplicons by SPRI clean-up using 1X Agencourt Ampure XP beads. The final concentration of amplicons is determined by qPCR with P5/P7 adapter primers compared against a standard curve in the Illumina qPCR Quantitation Kit, per manufacturer’s recommendation. Additionally, amplicon size and absence of primer dimer amplification should be confirmed prior to sequencing on the Fragment Analyzer, as discussed in Section 2.3.1 *step 7* above.
5. DNA sequencing can be performed on an Illumina MiSeq platform using MiSeq Reagent Kit v2 300 Cycles Nano Kit, or any other compatible platform.
6. For NGS data analysis we employ a freeware computational pipeline for CRISPR genome editing experiments, CRISPResso [21]. The plain text output from the computational analysis is used to generate graphical reports for visualizing *indel* profiles relative to the wild-type sequences.

### 2.4. Microinjection of murine zygotes and production of genetically altered mice

Mouse genome engineering using the CRISPR/Cas9 system has now become a standard approach [22]. The tool is routinely used for creating simple knockouts as well as knock-in mutations involving insertion of short sequences using ssODNs as HDR donors. Initial experimental protocols employed injection of DNA constructs that expressed sgRNAs and Cas9 nuclease [23,24], which were soon replaced by *in vitro* transcribed sgRNAs and Cas9 mRNA [25–27]. RNP complexes have been employed more recently and are considered to be more efficient for insertion of longer sequences, either using dsDNA [28] or by using ssDNA as donors [12]. The experimental protocol steps of assisted reproductive technologies (to generate a large number of zygotes by microinjection of genome editing components into and their subsequent transfer to pseudo-pregnant females) are typically performed in specialized core facility labs. Details of such experimental procedures, including identification of founders by genotyping, have been described in numerous publications [29–31]. Described below are steps for preparation of ctRNP complexes for mouse zygote microinjection. The ctRNP complexes are co-delivered with a repair template for insertion at the cleavage sites. We recently demonstrated that insertion frequency of repair DNA is higher if the template is supplied as ssDNA, instead of a dsDNA [7,12]. The methods for preparing long ssDNAs will be described in detail elsewhere (Miura et al., under review). Here we provide an example of generating a reporter knock-in mouse model by delivering ctRNP with long ssDNA via mouse zygote microinjection.

#### 2.4.1. ctRNP complex preparation for mouse zygote microinjection

The two RNA components (crRNA and tracrRNA) should be reconstituted in embryo grade injection buffer (10 mM Tris-HCl pH 7.5, 0.1 mM EDTA) and annealed to generate an active guide RNA, similar to methods described above (Section 2.1.1). Use embryo grade injection buffer for all subsequent dilutions. Use filter tips for pipetting and aseptic techniques. Follow IDT’s Resuspension Calculator formula at https://www.idtdna.com/Calc/resuspension to calculate the buffer volume. Note: like in many transgenic core laboratories, we use mass amounts (μg/μL) in our injection mix recipes, not molar concentrations. We typically use 5–20 ng/μL of guide RNA and 5–20 ng/μL of Alt-R™ S.p. Cas9 3NLS protein for mouse zygote microinjections. Other concentrations may also work.

1. Anneal Alt-R™ crRNA and tracrRNA. Prepare the guide RNA by mixing 5 μg of crRNA (5 μL of 1 μg/μL) and 10 μg of tracrRNA (10 μL of 1 μg/μL) and anneal in a thermocycler or water batch by heating to 95 °C for 5 min and cooling to room temperature.
2. Prepare ctRNP injection mix (100 μL volume). We use guide RNA (annealed crRNA and tracrRNA) and Alt-R™ S.p. Cas9 3NLS at 5–20 ng/μL final concentrations each. Calculate the volumes of guide RNA and Cas9 protein needed for 100 μL injection mix. First dilute guide RNA in a volume of ~80 μL injection buffer. Next, add Alt-R™ S.p. Cas9 3NLS protein to a final concentration of 5–20 ng/μL. Adjust the volume to 100 μL using injection buffer if donor DNA is not included in the injection mix (if donor DNA is included, adjust the volume after adding the donor DNA; see next step). Incubate at room temperature for 10–15 min to allow formation of ctRNP complexes. Notes: i) ctRNP complexes are formed during this incubation step; add donor DNA after the RNP complexes are generated, ii) since the Alt-R™ S.p. Cas9 3NLS protein is supplied at high concentrations, it may be more convenient to make an intermediary dilution (e.g., 200 ng/μL) and then dilute it to the final concentration of 20 ng/μL.
3. Add donor DNA to injection mix (optional). Suggested final concentration of donor DNA is 5–20 ng/μL. Adjust the final volume to 100 μL.
4. Centrifuge the injection mix at 21,000*g* for 5–10 min at room temperature. Take 80 μL from the top and pass through a Millipore filter (UFC30VV25, EMD Millipore, Billerica, MA, USA). Note: spinning and passing through filters is an additional precautionary step to eliminate any solid particles and prevent clogging of the microinjection needles.
5. Load the injection mix into needles and follow microinjection procedures described previously [29].

## 3. Results and discussion

### 3.1. Lipofection of Cas9 ctRNP complexes into HEK293 cells

**Fig. 3:**
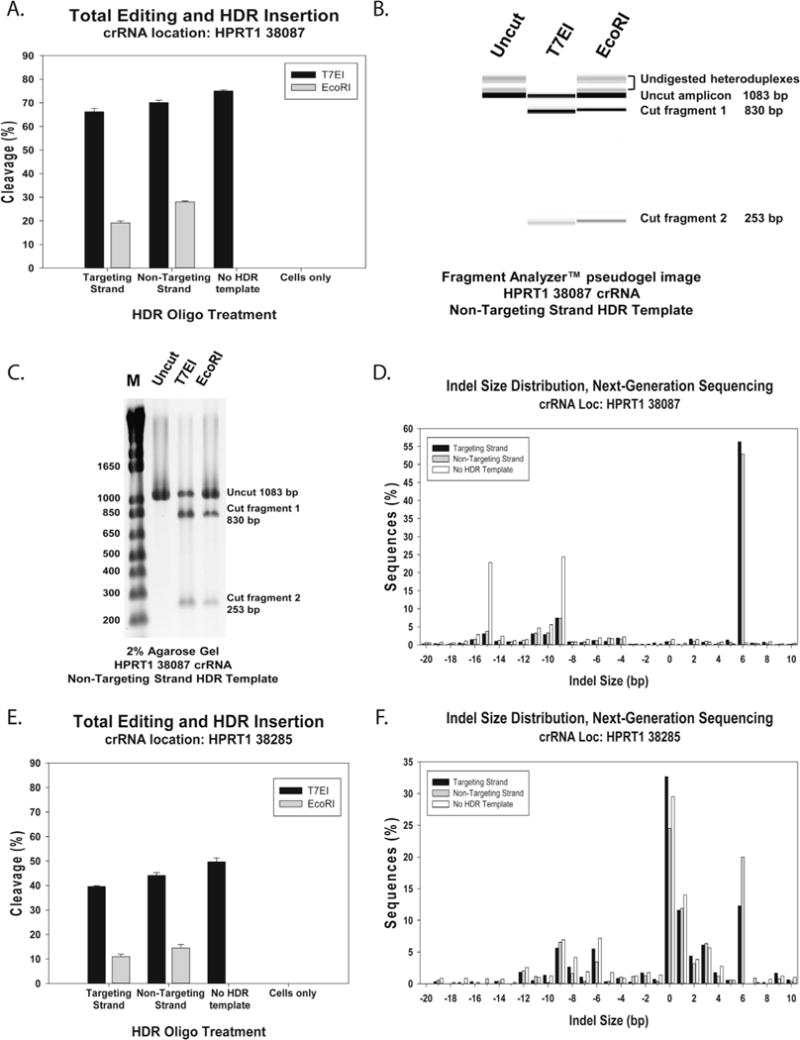
Lipofection of Cas9 ctRNP complexes into HEK293 cells. Cas9 ctRNP complexes with crRNAs specific for *HPRT1* sites 38087 and 38285 were delivered at 10 nM concentration into HEK293 cells with or without an HDR ssODN template to introduce a new EcoRI site. Plots depict genome editing events at the *HPRT1* locus 48 h post-transfection for total *indels* as assessed by a T7EI EMC assay or insertion of the EcoRI site by HDR: (A) editing efficiency following transfection of ctRNPs specific for *HPRT1* site 38087; (B) pseudogel image from the Fragment Analyzer™ showing the uncut site 38087 amplicon and amplicon cleavage products using the T7EI assay or EcoRI digestion; (C) agarose gel image showing the uncut site 38087 amplicon and amplicon cleavage products using the T7EI assay or EcoRI digestion; (D) NGS data showing specific *indel* frequency at *HPRT1* site 38087 with or without an EcoRI site HDR ssODN template; (E) editing efficiency following transfection of ctRNPs specific for *HPRT1* site 38285; (F) NGS data showing specific *indel* frequency at *HPRT1* site 38285 with or without an EcoRI site HDR ssODN template.

Lipofection is an effective approach to introduce Cas9 ctRNP into cells, so long as that cell type can be efficiently transfected with cationic lipids [32]. We find the cationic lipids Lipofectamine^®^ RNAiMAX and Lipofectamine^®^ CRISPRMAX™ (Thermo Fisher Scientific, Carlsbad, CA, USA) work well for lipofection of Cas9 ctRNP. A general rule of thumb holds that if siRNA can be transfected with RNAiMAX into a given cell line, lipofection of Cas9 ctRNP will also work in that cell line. Lipofection consumes one-tenth or less Cas9 protein and guide RNA than electroporation and does not require costly hardware or consumables, so use of lipofection methods can have cost advantages. Cas9 ctRNP complexes comprising crRNAs specific for two sites in the *HPRT1* gene (sites 38087 and 38285) were introduced into HEK293 cells using lipofection at 10 nM concentration, with or without a ssODN HDR template. The HDR templates were designed to introduce a 6 base EcoRI site directly at the Cas9 cleavage site. Cells were lysed 48 h after transfection and DNA was tested for genome editing at the *HPRT1* locus using a T7EI EMC assay and for insertion of the EcoRI HDR template by EcoRI digestion. Two HDR templates were tested, one using the “Targeting” and one using the “Non-Targeting” strand. The “Targeting Strand” is the DNA strand that is complementary to and bound by the crRNA protospacer. The “Non-Targeting Strand” is the free strand that is not associated with the crRNA after duplex unwinding and binding of the crRNA protospacer to the Targeting Strand. Results for site 38087 are summarized in Fig. 3A. The pseudogel image produced from capillary electrophoresis of the cleavage products at this site by the Fragment Analyzer™ is shown in Fig. 3B and the agarose gel visualization of T7EI and EcoRI cleavage products at this site is shown in Fig. 3C. Sequences of the *HPRT1*-specific crRNA protospacer domains, EMC assay PCR primers, and HDR templates are shown in Table 1. At site 38087, total genome editing was estimated to have occurred at 65–75% of the *HPRT1* loci in the cell population, with 20–30% of sites showing introduction of the EcoRI site by HDR. Results for site 38285 are summarized in Fig. 3E. Total genome editing was estimated to have occurred at 40–50% of the *HPRT1* loci in the cell population, with 10–15% of sites showing introduction of the EcoRI site by HDR. Note that the PCR primers used for the EMC and EcoRI assays are placed outside of the ssODNs homology arms used for HDR, so no false-positive artifacts can arise from amplification of any residual HDR template that might remain in the cells when DNA was harvested after 48 h incubation. The difference in efficiency between sites 38087 and 38285 is expected: different sites show differing levels of Cas9 cleavage and the relative accuracy of the T7EI assay varies with the spectrum of *indel* repair products produced. It is also common to see HDR efficiency vary from site to site, independent of the apparent efficiency of Cas9 cleavage (although more active crRNA sites often show higher HDR efficiency compared to low activity crRNA sites).

Genomic DNAs from the above genome editing experiments were evaluated using NGS with libraries prepared by the amplicon method (Section 2.3.4 above). DNA sequencing was performed on a MiSeq platform. The relative spectrum of *indels* produced are shown in Fig. 3D and F. The +6 *indel* peak corresponds with insertion of the EcoRI site and this peak is only seen in cultures where editing was performed with the ssODN HDR template. In Fig. 3D, the other major peaks seen at −9, and −15 are typically seen with CRISPR editing done at site 38087. They comprise the “fingerprint” associated with NHEJ repair in this sequence context and are better visualized when the HDR template is not employed [33]. Likewise, at site 38285, in Fig. 3F, the other major peaks seen at −9, −8, −6, +1, +2, and +3 are typically seen with CRISPR editing done at this site and comprise the “fingerprint” associated with NHEJ repair in this sequence context. NGS evaluation of genome editing at site 38087 showed that around 98% of genomic DNA strands in the cell population were altered with as high as 55% EcoRI site insertion by HDR, while the T7EI EMC assay showed at best 75% editing and 30% EcoRI site insertion by HDR. This highlights the inherent weakness of the EMC and other functional cleavage assays in evaluating genome editing results. Enzymatic methods usually underestimate changes at the DNA level. The T7EI enzyme does not cleave at all single-base mismatch or *indel* sites and so will not detect these events; it also cannot cleave a mutant DNA duplex if the same mutant top and bottom strands annealed during the final heteroduplex formation step since no mutation “bubble” will be present.

### 3.2. Electroporation of Cas9 ctRNP complexes into HEK293 cells

**Fig. 4:**
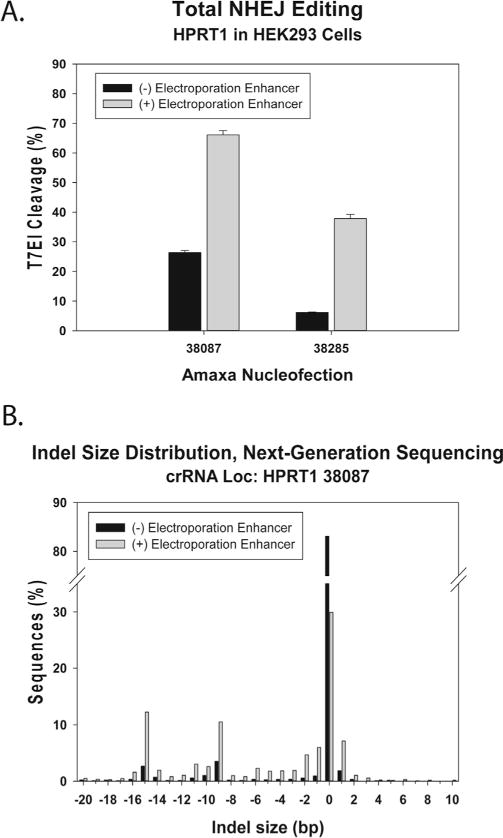
Electroporation of Cas9 ctRNP complexes into HEK293 cells. Cas9 ctRNP complexes with crRNAs specific for *HPRT1* sites 38087 and 38285 were delivered at 4 μM concentration into HEK293 cells with or without the Alt-R™ Cas9 Electroporation Enhancer ssODN using the Amaxa^®^ Nucleofector^®^. After 48 h incubation, genomic DNA was examined for editing at the *HPRT1* locus using: (A) T7EI EMC assay, or (B) NGS.

Electroporation is an effective method for introducing Cas9 ctRNP complexes into cells that are not amenable to lipofection. As an example of the relative efficiencies between lipofection and electroporation, HEK293 cells were electroporated using the Amaxa^®^ Nucleofector^®^ platform testing *indel* formation at the same two sites in the human *HPRT1* gene, 38087 and 38285. Cas9 ctRNP complexes were electroporated at 4 μM concentration, with and without addition of the Alt-R™ Cas9 Electroporation Enhancer (“NOE” reagent). After 48 h incubation, cells were lysed and genomic DNA was tested for genome editing at the *HPRT1* locus using the T7EI EMC assay. Results are shown in Fig. 4A. Sequences of the *HPRT1*-specific crRNA protospacer domains and EMC assay PCR primers are shown in Table 1. At site 38087, total genome editing was estimated to have occurred at around 70% of the *HPRT1* loci when using the Alt-R™ Cas9 Electroporation Enhancer, similar to the efficiency achieved using lipofection (Fig. 3A). Without the Alt-R™ Cas9 Electroporation Enhancer, efficiency was reduced to less than 30% cleavage. At site 38285, total genome editing was estimated to have occurred at around 40% of the *HPRT1* loci when using the Alt-R™ Cas9 Electroporation Enhancer, also similar to the efficiency achieved using lipofection (Fig. 3E). Without the Alt-R™ Cas9 Electroporation Enhancer, efficiency was reduced to less than 10% cleavage. Genomic DNAs from the cultures were amplified, Nextera™ libraries were prepared, and DNA sequencing performed on a MiSeq platform (Section 2.3.3). The relative spectrum of *indels* produced at site 38087 are shown in Fig. 4B. The major peaks seen (−9, and −15) again reflect the “fingerprint” associated with once NHEJ repair at this site and the pattern is very similar to that seen previously at the same site when the ctRNP was delivered using lipofection (Fig. 3D). The fingerprint pattern is the same with or without the Alt-R™ Cas9 Electroporation Enhancer, only the magnitude of *indel* frequency varies.

The Alt-R™ Cas9 Electroporation Enhancer is a ssODN with no sequence homology to the human, mouse, or rat genomes. The benefits of using “non-homologous oligonucleotide enhancement” (“NOE”) have been reported previously and were proposed to result from diverting cells towards error-prone repair [14]. We find that use of the Alt-R™ Cas9 Electroporation Enhancer ssODN often improves efficiency of NHEJ gene disruption in Cas9 ctRNP genome editing applications, but only when electroporation is employed; we have seen no benefit when adding the Alt-R™ Cas9 Electroporation Enhancer ssODN if the ctRNP is delivered using lipofection (data not shown). The magnitude of improvement varies widely between cell types and the electroporation protocols employed (Table 2). Although it is theoretically possible that the ssODN might participate in break repair events, we have not seen evidence for insertion of the Alt-R™ Cas9 Electroporation Enhancer ssODN into the repair site by NGS (Fig. 4B). Using long amplicons with Nextera™ library preparation, it is possible to identify long inserts of 100–200 base length by NGS if the Alt-R™ Cas9 Electroporation Enhancer ssODN was inserted at the break point, which would not be as easily seen using direct amplicon methods. Insertions of this kind were not seen. Further, insertions are not increased in frequency relative to deletion events after break repair when ctRNP delivery is performed with the Alt-R™ Cas9 Electroporation Enhancer than without its use (data not shown). It was previously reported that stimulation of repair pathways using non-homologous ssODN reagents altered the spectrum of repair product seen after healing of Cas9-induced dsDNA breaks [14]. Our limited NGS results do not show evidence for an altered spectrum of repair outcomes and simply show higher levels of indel formation with a similar “fingerprint”, comparing electroporations done with or without the “NOE” (Alt-R™ Cas9 Electroporation Enhancer) reagent (Fig. 4B, and data not shown). When adding a ssODN HDR template to the electroporation mixture, we recommend omitting the Alt-R™ Cas9 Electroporation Enhancer as the HDR nucleic acid adequately serves the same function and no additional benefit is seen with its use.

### 3.3. Electroporation of Cas9 ctRNP complexes into Jurkat cells

**Fig. 5:**
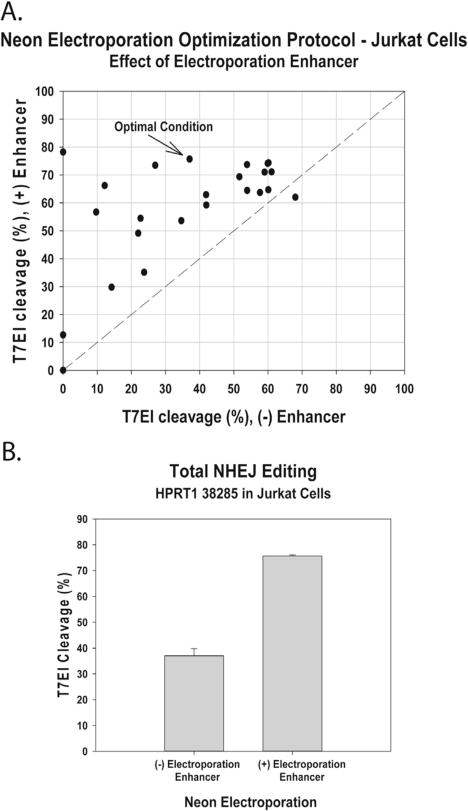
Optimization of electroporation of Cas9 ctRNP complexes into Jurkat T-cells. Cas9 ctRNP complexes specific for *HPRT1* site 38087 were delivered at 1.5 μM concentration into Jurkat T-cells with or without the Alt-R™ Cas9 Electroporation Enhancer ssODN using the Neon™ Transfection System under 24 different conditions (Table 2). Plots depict genome editing events at the *HPRT1* locus 72 h post-transfection for total *indels* as assessed by the T7EI EMC assay: (A) the editing efficiency for each electroporation condition with the Alt-R™ Cas9 Electroporation Enhancer is measured on the Y-axis and without the Alt-R™ Cas9 Electroporation Enhancer is measured on the X-axis; (B) editing efficiencies for the best condition (condition #24, Table 2), with and without Electroporation Enhancer.

Systematic optimization of electroporation protocols can dramatically improve Cas9 ctRNP delivery and cell viability. Full optimization is convenient to perform using the *Neon™ Transfection System*, which permits individual variation of all parameters (voltage, pulse width, and number of pulses). Cas9 ctRNP has been efficiently delivered into human primary T-cells using the *Neon™ Transfection System* [34]. We performed a full optimization matrix in Jurkat T-cells comparing the efficiency of *indel* formation as assessed by the T7EI EMC assay at site *HPRT1* 38285 using 1.5 μM Cas9 ctRNP with or without 1.8 μM Alt-R™ Cas9 Electroporation Enhancer. The full experiment is shown in Table 2, where a wide range of genome editing efficiencies and cell viabilities were seen over the range of conditions tested. Several conditions resulted in >70% editing with acceptable cell viability. The relative genome editing efficiencies (*indel* rates detected by T7EI EMC assay) were plotted comparing electroporation with or without the optional Alt-R™ Cas9 Electroporation Enhancer in Fig. 5A. Results were superior using the Alt-R™ Cas9 Electroporation Enhancer in 23/24 of the conditions tested. Results for the ‘optimal condition’, having both very high editing efficiency and high cell viability, are highlighted in Fig. 5B. Systematic optimization of electroporation conditions is beneficial whenever a cell type will be repeatedly used in genome editing experiments or if having the highest possible efficiency and cell viability is critical to experimental results.

### 3.4. Microinjection of murine zygotes with ctRNP complexes

The ctRNP CRISPR complexes were seen to have higher efficiency of cleavage and higher HDR rates compared to other methods (sgRNA + Cas9 mRNA or plasmids expressing sgRNAs and Cas9) [9,35,36], and use of RNP methods show reduced off-target effects [4,5,10]. The observed higher efficiency most likely relates to the immediate availability of functional CRISPR RNP complexes following microinjection, whereas the other methods all face a time delay in forming an active RNP complex inside the zygotes while Cas9 transcription and/or translation occurs from the injected templates. We have also noticed higher knock-in efficiency of donor templates, using either ssODNs (data not shown) or long ssDNAs [7] as donors. Even though the ctRNP approach is highly efficient for mouse genome editing, this method does not offer solutions to the general pitfalls of using CRISPR systems, such as mosaicism and off-target cleavage effects. However, such issues are not a major concern for animal genome editing considering that (a) the majority of mosaic founders typically produce germ line transmitted offspring and (b) the off-target cleavages, if any, can be segregated through breeding the mutant lines [37].

**Fig. 6:**
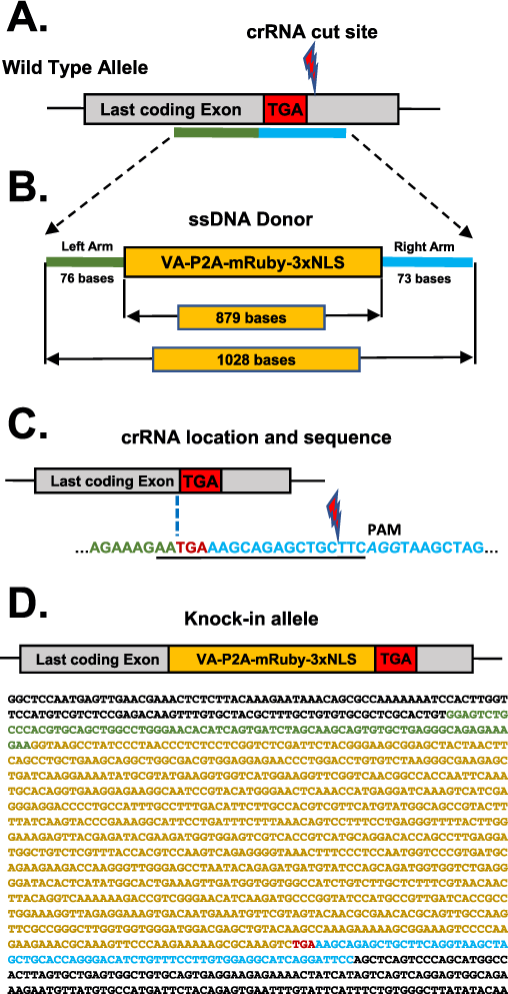
Generation of *DDC-mRuby* knock-in allele. (A) Schematics of the last coding exon of *DDC* gene and the knock-in ssDNA donor homology arm sites. The stop codon (TGA) is marked, the left and right homology regions are shown in green and blue bars respectively (not to scale). (B) Schematic of the ssDNA donor showing the lengths of the homology arms and the knock-in cassette. (C) Schematic showing the location of the closet available guide RNA to the stop codon. The crRNA guide sequence is underlined and the NGG PAM sequence is italicized. Note that the desired insertion site (which is immediately before the stop codon; marked with a dotted line) and the Cas9 cleavage site (indicated) are separated by 15 bases. The homology arm sequences of the donor sequence are designed to achieve insertion at the desired insertion site (15 bases upstream of the cleavage site). The corresponding colors of the left- (green) and right- (blue) homology arms are retained within the guide sequence. (D) Schematic of the knock-in allele and its sequence. Corresponding colors are used for the rectangles in the schematic (above) and for the sequence in the text (below) with green indicating left homology arm, yellow indicated insert sequence, red indicating the stop codon, and blue indicating the right homology arm.

**Fig. 7:**
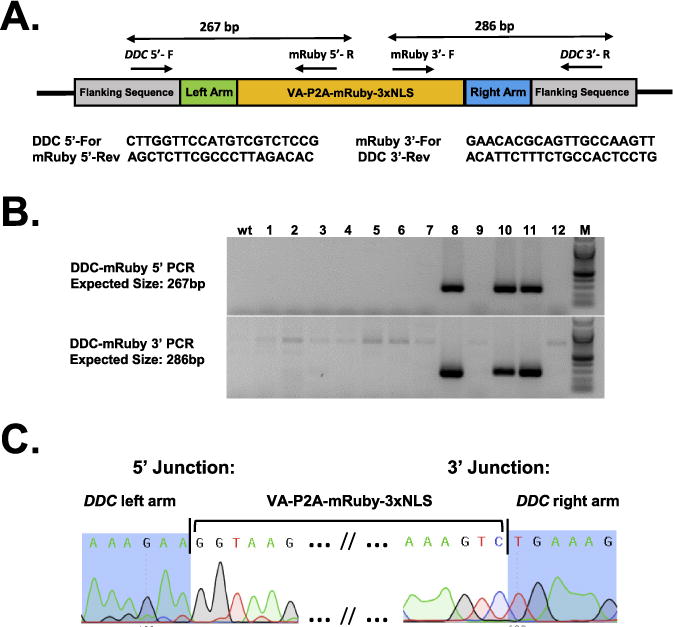
Sequence confirmation of *DDC-mRuby* knock-in allele. (A) Schematic showing the *DDC-VA-P2A-mRuby-3X NLS* knock-in allele and the location of genotyping PCR primers. Two PCR reactions, one each for 5′ and 3′ junction of the insert, were used for assessing the insertions. The PCR amplicon sizes are shown above the schematic and the primer sequences below the schematic. (B) Genotyping agarose gel images showing correct insertions at 5′ (top panel) and 3′ (bottom panel). Animals #8, #10 and #11 show expected amplicons for both the junction PCRs. Sequencing of the PCR products showed that animals #8 and #10 had indels or mutations whereas the animal #11 had precise insertions at both the junctions without any mutations. (C) Sequencing of the insertion junctions of the animal #11 showing precise insertion of the cassette at the desired site (immediately before the stop codon); compare with the sequences shown in Fig. 6C and D.

We recently described a strategy called *Easi-*CRISPR (*E*fficient *a*dditions with *s*sDNA *i*nserts-CRISPR) that employs the use of long ssDNAs as repair donors, in combination with ctRNP complexes, for creating knock-in and conditional knockout animal models [7]. Here we demonstrate an example of generating a reporter cassette knock-in allele using the *Easi-*CRISPR strategy. The knock-in cassette consisted of a 0.9 kb cassette encoding VA-P2A-mRuby 3xNLS inserted immediately before the stop codon of the *DDC* gene (VA, immunoaffinity tag; P2A; viral protease for post-translational cleavage of the reporter protein from the DDC protein; 3XNLS; nuclear localization signal). A Cas9 PAM motif guide site search was performed using the sequence surrounding the termination codon of the *DDC* gene. The closest available guide cleaves 15 nucleotides downstream of the desired insertion site (immediately prior to the stop codon). The ssDNA donor DNA schematics, guide location and sequences are shown in Figs. 6A–C. The genotyping schematics and the results are shown in Figs. 7A–C. The ctRNP complex, consisting of the crRNA and equimolar ratio of tracrRNA, were prepared (as described in Section 2.4.1) and microinjected into C57BL/6 strain-derived mouse zygotes along with the 1028 base long ssDNA donor DNA, which was synthesized as described previously [12]. The concentrations of ctRNPs used were 5 ng/μL of the guide RNA, 5 ng/μL of Cas9 protein and 10 ng/μL of the ssDNA donor. The ctRNP and ssDNA donor mix was injected into 41 zygotes; 38 of these zygotes were transferred into recipient females following standard protocols [29]. Genotyping of the 12 pups born show that animals #8, #10 and #11 contained insertion of the knock-in cassette. Of these, animal #11 had correct insertion of the cassette at the desired insertion site whereas animals #8 and #10 had imperfect insertions with *indels* near the insertion sites or mutations in the cassette.

## 4. Concluding remarks

During the past four years, a variety of methods to introduce guide RNAs and Cas9 nuclease into cells have been used for genome editing experiments. The most recent approach employs pre-formed complete ribonucleoprotein (RNP) complexes and does not require expression of any of these elements in the host cell. Of the two versions of RNP complexes, the combination of crRNA, tracrRNA and Cas9 protein offer several advantages: (a) the tracrRNA is a universal component and can be produced in bulk so that a single stock can be used many times with different crRNAs; (b) the crRNA (the component that confers sequence specificity) is short, so it can be synthesized very quickly and at lower costs than required for chemical manufacture of a long sgRNA; (c) since the shorter component crRNAs and tracrRNA can be easily chemically synthesized, chemical modifications can be incorporated to enhance the stability and reduce immunogenicity of the foreign RNAs in cells and; (d) different levels of chemical modification (low, medium or high) can be introduced as needed for different experimental needs (Collingwood et al., manuscript in preparation). Because of these advantages, the ctRNP-based CRISPR tools offer the simplest and most efficient protocols for genome editing in cells and zygotes.

## Figures and Tables

**Table 1:** Oligonucleotide sequences.

Oligonucleotide	Sequence
HPRT1-38087 protospacer	aauuauggggauuacuagga
HPRT1-38285 protospacer	cuuauauccaacacuucgug
HPRT1-For1	AAGAATGTTGTGATAAAAGGTGATGCT
HPRT1-Rev1	ACACATCCATGGGACTTCTGCCTC
HPRT1-For2	CTTCAGGTTCCAGGTGATCA
HPRT1-Rev2	CTAGACTACAGCTTTATGTGACT
HPRT1 38087 For PCR1	ACACTCTTTCCCTACACGACGCTCTTCCGATCTCTTCAGGTTCCAGGTGATCA
HPRT1 38087 Rev PCR1	GTGACTGGAGTTCAGACGTGTGCTCTTCCGATCTTCATCCGTGCTGAGTGTAC
HPRT1 38285 For PCR1	ACACTCTTTCCCTACACGACGCTCTTCCGATCTGCCCTGTAGTCTCTCTGTATG
HPRT1 38285 Rev PCR1	GTGACTGGAGTTCAGACGTGTGCTCTTCCGATCCTGGCAAATGTGCCTCTCTA
P5 Primer For PCR2	AATGATACGGCGACCACCGAGATCTACACNNNNNNNNACACTCTTTCCCTACACGACGCTCT
P7 Primer Rev PCR2	CAAGCAGAAGACGGCATACGAGATNNNNNNNNGTGACTGGAGTTCAGACGTGTGC
HDR-38087-T	CTGTAGTGTCAACTCATTGCTGCCCCTTCCGAATTCTAGTAATCCCCATAATTTAGCTCTCCATTT
HDR-38087-NT	AAATGGAGAGCTAAATTATGGGGATTACTAGAATTCGGAAGGGGCAGCAATGAGTTGACACTACAG
HDR-38285-T	TTAACAGCTTGCTGGTGAAAAGGACCCCACGAATTCGAAGTGTTGGATATAAGCCAGACTGTAAGT
HDR-38285-NT	ACTTACAGTCTGGCTTATATCCAACACTTCGAATTCGTGGGGTCCTTTTCACCAGCAAGCTGTTAA

Nucleic acid sequences are shown 5′-3′. RNA residues are lowercase, DNA residues are uppercase. PCR primers For1/Rev1 are used for the T7EI EMC assay and For2/Rev2 for NGS Nextera™ library preparation. PCR primers HPRT1 38087 For PCR1, HPRT1 38087 Rev PCR1, HPRT1 38285 For PCR1, and HPRT1 38285 Rev PCR1 are used for the first step of amplicon sequencing library preparation at the respective crRNA sites. PCR primers P5 Primer For PCR2 and P7 Primer Rev PCR2 are used for the second step of amplicon sequencing library preparation. T = Targeting strand, NT = Non-Targeting strand. The “Targeting Strand” is the DNA strand that is complementary to and bound by the crRNA protospacer. The “Non-Targeting Strand” is the free strand that is not associated with the crRNA after duplex unwinding and binding of the crRNA protospacer to the Targeting Strand.

**Table 2:** Optimization of electroporation conditions for Jurkat cells using the Neon platform.

	*Editing Efficiency (%)*		*SD (%)*		*Cell Density*		*Pulse*		
	− EE	+ EE	− EE	+ EE	− EE	+ EE	Voltage (V)	Width (ms)	Number

Prot_01	0.0	0.0	0.0	0.0	3	4	0	1	1

Prot_02	53.9	64.4	2.7	1.8	2	3	1400	20	1
Prot_03	59.0	71.0	0.5	4.5	2	3	1500	20	1
Prot_04	0.0	78.2	0.0	1.3	3	1	1600	20	1
Prot_05	60.1	64.7	3.1	13.2	2	2	1700	20	1

Prot_06	22.0	49.1	0.6	16.3	1	4	1100	30	1
Prot_07	41.9	62.9	3.2	6.8	3	4	1200	30	1
Prot_08	57.7	63.7	0.9	15.2	2	4	1300	30	1
Prot_09	60.2	74.4	2.4	3.1	3	2	1400	30	1

Prot_10	14.2	29.8	1.4	0.4	3	2	1000	40	1
Prot_11	34.6	53.6	0.9	0.1	4	1	1100	40	1
Prot_12	27.0	73.5	1.7	3.8	2	2	1200	40	1

Prot_13	22.7	54.5	1.3	4.6	2	2	1100	20	2
Prot_14	51.6	69.4	1.9	0.8	2	2	1200	20	2
Prot_15	59.9	74.2	3.0	0.7	2	2	1300	20	2
Prot_16	68.0	62.0	2.6	11.2	3	4	1400	20	2

Prot_17	0.0	12.7	0.0	0.3	4	4	850	30	2
Prot_18	23.7	35.1	0.9	2.0	3	2	950	30	2
Prot_19	9.7	56.7	0.6	3.1	3	3	1050	30	2
Prot_20	53.9	73.7	3.6	7.2	3	2	1150	30	2

Prot_21	41.9	59.2	1.4	1.4	3	4	1300	10	3
Prot_22	12.2	66.2	1.1	0.2	3	4	1400	10	3
Prot_23	61.1	71.1	1.7	2.2	3	2	1500	10	3
Prot_24 ✓	37.0	75.7	2.8	0.4	2	4	1600	10	3

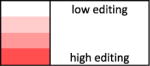


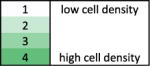

EE = Electroporation Enhancer

SD = Standard Deviation

Low cell density correlates with low cell viability and high cell density correlates with high cell viability post electroporation.
